# Prediction of Core Body Temperature Based on Skin Temperature, Heat Flux, and Heart Rate Under Different Exercise and Clothing Conditions in the Heat in Young Adult Males

**DOI:** 10.3389/fphys.2018.01780

**Published:** 2018-12-10

**Authors:** Patrick Eggenberger, Braid A. MacRae, Shelley Kemp, Michael Bürgisser, René M. Rossi, Simon Annaheim

**Affiliations:** ^1^Empa, Swiss Federal Laboratories for Materials Science and Technology, Laboratory for Biomimetic Membranes and Textiles, St. Gallen, Switzerland; ^2^Institute of Human Movement Sciences and Sport, Department of Health Sciences and Technology, ETH Zurich, Zurich, Switzerland

**Keywords:** core body temperature, rectal temperature, skin temperature, heat flux, heart rate, exercise, heat strain, prediction model

## Abstract

Non-invasive, multi-parameter methods to estimate core body temperature offer several advantages for monitoring thermal strain, although further work is required to identify the most relevant predictor measures. This study aimed to compare the validity of an existing and two novel multi-parameter rectal temperature prediction models. Thirteen healthy male participants (age 30.9 ± 5.4 years) performed two experimental sessions. The experimental procedure comprised 15 min baseline seated rest (23.2 ± 0.3°C, 24.5 ± 1.6% relative humidity), followed by 15 min seated rest and cycling in a climatic chamber (35.4 ± 0.2°C, 56.5 ± 3.9% relative humidity; to +1.5°C or maximally 38.5°C rectal temperature, duration 20–60 min), with a final 30 min seated rest outside the chamber. In session 1, participants exercised at 75% of their heart rate maximum (HR max) and wore light athletic clothing (t-shirt and shorts), while in session 2, participants exercised at 50% HR max, wearing protective firefighter clothing (jacket and trousers). The first new prediction model, comprising the input of 18 non-invasive measures, i.e., insulated and non-insulated skin temperature, heat flux, and heart rate (“Max-Input Model”, standard error of the estimate [SEE] = 0.28°C, *R^2^* = 0.70), did not exceed the predictive power of a previously reported model which included six measures and no insulated skin temperatures (SEE = 0.28°C, *R^2^* = 0.71). Moreover, a second new prediction model that contained only the two most relevant parameters (heart rate and insulated skin temperature at the scapula) performed similarly (“Min-Input Model”, SEE = 0.29, *R^2^* = 0.68). In conclusion, the “Min-Input Model” provided comparable validity and superior practicality (only two measurement parameters) for estimating rectal temperature versus two other models requiring six or more input measures.

## Introduction

Occupational or sports-related physical activities in the heat constitute major challenges to thermal homeostasis (Taylor, 2006; Gonzalez-Alonso et al., 2008; Nybo, 2008). Accurate detection of heat strain is important because an excessive increase in core body temperature adversely affects physical (Cheuvront et al., 2010) and cognitive performance (Nybo, 2008), places greater demands on the cardiovascular system, and can lead to hyperthermia and organ failure. Thus, early detection of excessive perturbations of core body temperature is advantageous for individuals subjected to extreme conditions and facilitates early implementation of interventional cooling strategies to avoid exertional heat stroke (Epstein and Roberts, 2011).

Information about core body temperature is central to the quantification of heat strain. However, common measurement procedures (e.g., esophageal, rectal, and gastrointestinal temperature) can lack practicality outside of the laboratory environment, particularly during prolonged use. Thus, valid and non-invasive surrogate measures for monitoring heat strain are required. Previous studies presented mathematical prediction models to estimate core body temperature using non-invasive physiological measurement parameters, including skin temperatures and heat fluxes at various body sites, heart rate, breathing frequency, accelerometry, and environmental climatic variables such as air temperature, radiant temperature, relative humidity, and wind speed (Buller et al., 2008, 2011, 2013, 2015; Yokota et al., 2008, 2012; Niedermann et al., 2014b; Richmond et al., 2015; Mazgaoker et al., 2017; Laxminarayan et al., 2018; Looney et al., 2018; Welles et al., 2018). However, further research is warranted to identify the most relevant predictor variables and to examine the general validity of these models.

Skin temperature was found to provide relevant information for estimating core body temperature, although the use of single skin temperatures underestimated rectal temperature (Mendt et al., 2017). In addition to conventional skin temperature measurements, the use of insulated skin temperatures might improve prediction accuracy. Thereby, insulating the temperature sensors serves to mitigate environmental effects and prevent local heat loss at the measured site and, in this way, may better reflect the temperature of deeper tissues (Richmond et al., 2015). Insulated skin temperatures have been used to estimate muscle tissue temperature (Brajkovic et al., 2006) and core body temperature over bony (spine; Richmond et al., 2013, 2015) or arterial sites (carotid; Jay et al., 2013). Nevertheless, in these studies, only one site was used, while the use of several sites together may improve a model’s predictive power.

Niedermann et al. (2014b) suggested that heat flux measurements need to be incorporated for an accurate prediction of rectal temperature. They used six parameters in their model, including three skin temperature and two heat flux measurement sites, as well as heart rate. However, as their model underestimated core body (rectal) temperature for prolonged applications, they proposed to include additional measurement sites, particularly for heat flux measurements. Heat flux was a main predictor of core body temperature also in other studies (Buller et al., 2011; Xu et al., 2013; Welles et al., 2018), and was applied to investigate body heat balance under various conditions (Flouris and Cheung, 2009; Kenny et al., 2009; Basset et al., 2011). Although a prediction model comprising multiple measurement parameters at various body sites might have advantages in terms of validity, a model incorporating as few parameters as possible is consistent with the concept of parsimony and has practical measurement advantages [e.g., fewer sensors required lessens issues with loss of sensor signals, conflicts between sensors, or other interferences that affect signal quality (Yokota et al., 2012; Looney et al., 2018)].

Therefore, the *aims* of this study were (1) to define the validity of a novel multi-parameter model, comprising 18 non-invasive measurements at various body sites to predict rectal temperature under two different exercise and clothing conditions in a hot and moderately humid environment, as well as at rest at normal room temperature; (2) to compare the novel prediction model, which includes additional measures of heat flux and insulated skin temperature at various body sites, with a previously published model (Niedermann et al., 2014b); and (3) to characterize the extent to which a stepwise reduction of the number of measured parameters, with the goal to improve practicality, affects the validity of the initial prediction model.

We *hypothesized*, that (1) the proposed model achieves the acceptance criterion of < 0.5°C deviation between the predicted and measured rectal temperature (Gunga et al., 2009; Yokota et al., 2012; Niedermann et al., 2014b); (2) the addition of heat flux and insulated skin temperature measures improves prediction validity in comparison with our previous model (Niedermann et al., 2014b); and (3) the validity of an adapted/reduced model, including fewer parameters for optimized practicality, still is in an acceptable range (i.e., deviation between predicted and measured rectal temperature < 0.5°C).

## Materials and Methods

### Study Design and Participants

This study comprised one preliminary session to assess participants’ characteristics and two experimental sessions to collect data for establishing models for the non-invasive prediction of rectal temperature. Data collection was performed at the Swiss Federal Laboratories for Materials Science and Technology, Empa, St. Gallen, Switzerland. This study was carried out in accordance with the recommendations of the Human Research Act and the Human Research Ordinance (The Swiss Federal Council, 2013), and the principles of Good Clinical Practice with written informed consent from all subjects. The study protocol was approved by the ethics committee of Eastern Switzerland (Project-ID: 2017-01376, EKOS 17/129, SNCTP000002592) and performed in accordance with the Declaration of Helsinki.

The participants were recruited from September to November 2017 through advertisements at the city’s firefighter association, the local universities, and at fitness centers; testing sessions were performed between November 2017 and March 2018. For eligibility, participants had to be male, between 18 and 45 years of age, apparently healthy (passed health screening questionnaire), non-smokers, physically active (i.e., regularly for at least twice a week), and sign informed consent. Because factors like age, gender, and health status may influence thermoregulatory response, a homogeneous group, i.e., adult active males, was used in this study (Kaciuba-Uscilko and Grucza, 2001; Gagnon and Kenny, 2012; Cheuvront, 2014; Cramer and Jay, 2014).

### Experimental Protocol

The preliminary session comprised the participants’ information and screening with a pre-activity health questionnaire modified from Thomas et al. (1992), assessments of body weight and height, % body fat, as well as an incremental maximal exercise test on the cycling ergometer to determine maximal heart rate and exercise intensity at 50 and 75% heart rate, respectively. Initial resistance was set at 70 W and was increased by 30 W every 2 min until volitional fatigue of the participant. Participants were asked not to consume any alcohol or caffeine 12 h prior to the sessions.

**FIGURE 1:**
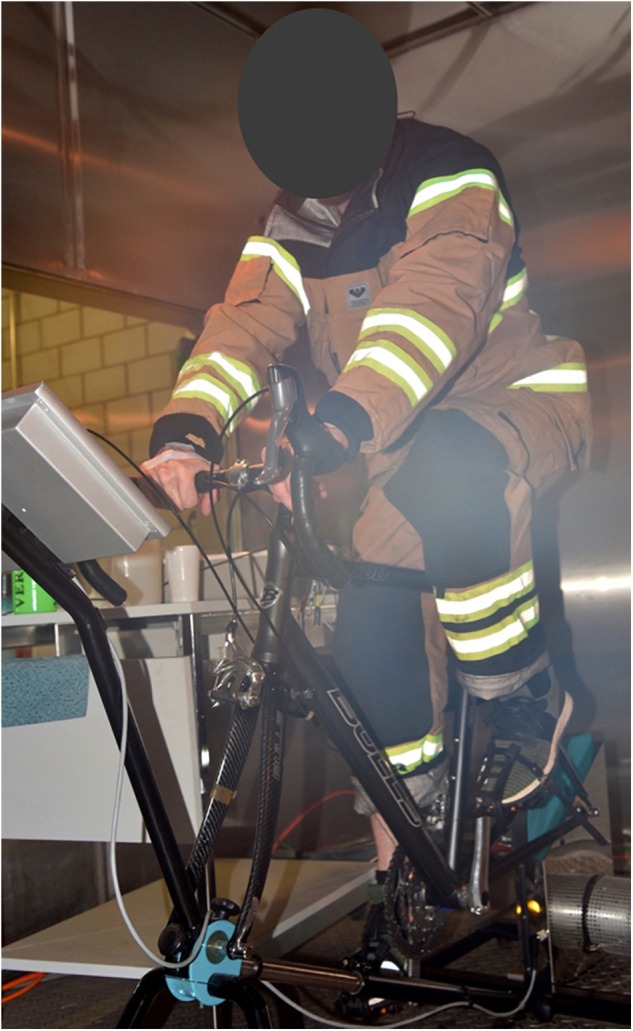
Participant cycling at 50% HR max, wearing protective firefighter clothing during heat session 2.

**FIGURE 2:**
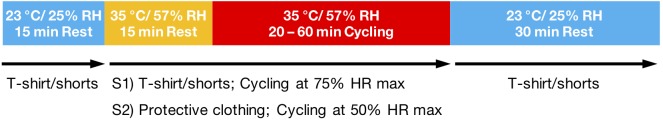
Experimental procedures for heat sessions 1 and 2. HR max, maximal heart rate; RH, relative humidity; S1, heat session 1; S2, heat session 2.

During the experimental sessions, heat strain was induced by exposure to a combination of environmental heat and physical activity. The two experimental sessions (heat sessions 1 and 2) were completed at the same time of day and at least 7 days apart to mitigate acclimation after session 1. The sessions differed in the intensity of exercise and the thermal insulation of the clothing worn, which were selected to enable the development of a prediction model for rectal temperature applicable under various conditions. In heat session 1, participants exercised at 75% of their heart rate maximum (HR max) and wore light athletic clothing (t-shirt and shorts), while in heat session 2, participants exercised at 50% HR max, wearing protective firefighter clothing (jacket and trousers, worn in addition to the t-shirt and shorts from session 1; Viking Life-saving Equipment, Denmark, fulfilling performance requirements according to EN 469:2005, level 2; Figure 1). The environmental conditions in the climatic chamber were 35.4 ± 0.2°C, 56.5 ± 3.9% relative humidity (RH), and ∼0.5 m/s air velocity. Outside the chamber, climatic conditions were 23.2 ± 0.3°C and 24.5 ± 1.6% RH. The experimental procedure is depicted in Figure 2 and started with a 15-min baseline seated rest outside the chamber, followed by 15 min seated rest and between 20 – 60 min cycling inside the chamber (exercise was stopped either when rectal temperature increased ≥ 1.5°C above baseline or ≥ 38.5°C (ACGIH, 2017; Methner and Eisenberg, 2018), at volitional fatigue, or after maximally 75 min), and ended with another period of 30 min seated rest outside the chamber. Participants were given 0.2 l of drinking water every 15 min, the first as they entered the climatic chamber. The water was tempered for about 30 min inside the chamber prior to the beginning of the experiment.

### Measurements

#### Primary Outcome Measures

**FIGURE 3:**
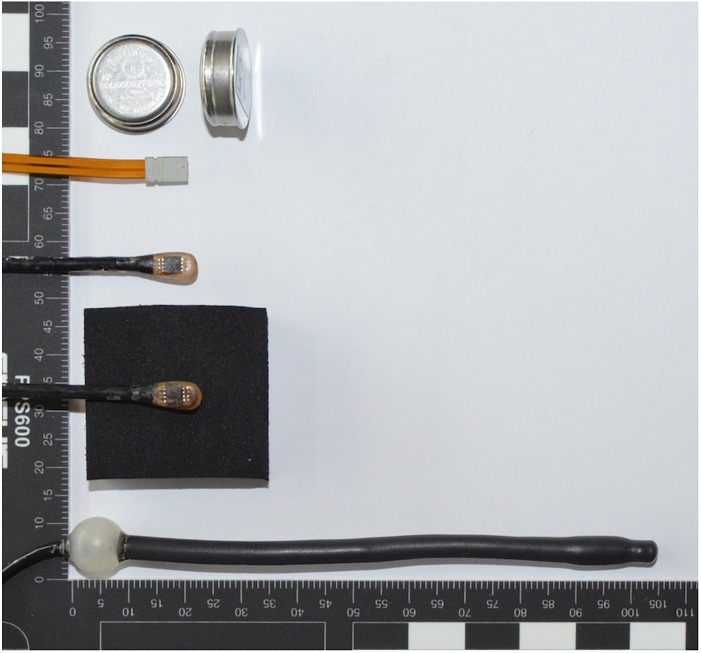
Temperature and heat flux sensors used in the study (scale in mm). From top down: iButton, heat flux sensor, non-insulated temperature sensor, insulated temperature sensor (side in contact with the skin is facing upward), and rectal temperature sensor.

*Rectal temperature* served as the reference measurement and was measured using a sterile rectal thermometer (type DS18B20, MSR Electronics, Switzerland; 4 – 6.5 mm diameter, 104 mm length; polyolefin cover) 10 cm past the anal sphincter. The following parameters were measured to establish a predictive model for rectal temperature: *insulated skin temperature* using MSR thermistors (type DS18B20, MSR Electronics, Switzerland; 2 mm × 5 mm × 9 mm; polyolefin cover) attached to the sternum (upper part), scapula (inferior angle), ribs (no. 8/9), and radial artery; *uninsulated skin temperature* at the same body sites and additionally, using iButtons (type DS1922L, Maxim Integrated, United States; ∼16 mm diameter, 6 mm height; stainless steel outer), on the forehead (center), upper arm (1/2 distance acromion – radial head), forearm (1/2 distance olecranon – ulnar head), back of the hand (center), thigh (1/2 distance inguinal crease – patella margo superior), calf (at maximal circumference); *skin heat flux* on the sternum (upper part), scapula (inferior angle), rib (no. 8/9) using gSKIN heat flux sensors (type XM 26 9C, greenTEG, Switzerland; 0.5 mm × 4.4 mm × 4.4 mm; aluminum sensing area); *heart rate* from 2-lead ECG data acquisition via ECG chest belt (Unico swiss tex, Switzerland) and Faros loggers (Bittium Biosignals, Finland). The temperature and heat flux sensors are shown in Figure 3. All data were recorded at 0.1 Hz sampling rate.

Temperature sensors (MSR thermistors, iButtons) were calibrated at steady states between 15°C and 40°C (5°C intervals) using a calibration chamber (OptiCal, Michell Instruments, United Kingdom). The heat flux sensors were calibrated using a guarded hotplate apparatus in a double plate configuration which generated a heat flux across the sample over the range of 50 – 180 W/m^2^ (12 steps). The heat flux sensors were calibrated in combination with the tape attachment (Transpore surgical tape 1527-2, 3M, United States) (Niedermann et al., 2014a).

The skin was cleaned with alcohol wipes and shaved where necessary. Temperature sensors were attached using a single layer of Hypafix tape (BSN Medical, Germany) and heat flux sensors were attached using a single layer of Transpore tape. Insulated skin temperature sensors were insulated using a 10 mm × 30 mm × 30 mm piece of closed cell foam (density = 180.38 kg/m^3^) and attached with Hypafix tape.

#### Secondary Outcome Measures

Participants’ characteristics included measures of standing *body height, body weight* (ID5 Multi range scale, Mettler Toledo, Switzerland), and *% body fat* calculated based on the sum of three skin folds (chest, abdomen, and thigh) assessed with a Harpenden skin-fold caliper (Baty International, United Kingdom) and the regression equation from Jackson and Pollock (1978). Additionally, body weight (wearing only sport shorts) was measured before and after heat sessions 1 and 2 in order to estimate *sweat loss*. During the maximal exercise test in the preliminary session, *heart rate* was measured using a Polar RS800CX heart rate monitor (Polar Electro, Finland).

### Data Processing

For the heart rate data, a moving average filter that included a 10-min range (=60 time points) before the time point of prediction was applied in order to prevent rapid fluctuations in the prediction model and to better reflect gradual increase of rectal temperature with increasing metabolic rate. Calculated calibration factors were applied to the temperature and heat flux data. Heat flux data were filtered applying a digital zero-phase second order low-pass Butterworth filter with a cutoff frequency of 0.2 Hz. Furthermore, heat flux data were visually inspected for artifacts and signal sections above 300 W/m^2^ and below -100 W/m^2^ were removed. Finally, a moving average filter including the values of the previous 6.7 min (=40 time points) was applied to consider the history of heat gain or heat loss which is measured with heat flux. No filtering or smoothing of the temperature data was applied as no artifacts were evident.

Cross-correlation analysis (maximum number of lags set at 200) was used to identify 12.5 min as the average delay of peak rectal temperature compared to the peak skin temperatures and heart rate (number of lags = 75). Therefore, the first 12.5 min of the rectal temperature data were removed and the time delay was taken into account for rectal temperature prediction, such that the value for rectal temperature at 12.5 min corresponded with the values of the skin temperatures and heart rate at the beginning of the experiment.

In the cases where data were missing due to sensor malfunctioning, detachment, or artifacts, all other measured parameters at the same time points were excluded from further analysis in order to obtain complete data sets at any remaining time point of the experiment. After this procedure, outliers were identified and removed as defined by Mahalanobis distance > 42.31 (according to χ^2^ critical values for *df* = 18 and *p* = 0.001) and by standardized residuals < -3 or >3 (Field, 2018). Matlab 2017b, IBM SPSS Statistics 25, and Microsoft Excel software were used for data processing.

### Statistical Analysis

The whole data set was split by randomly dividing the participants into two groups for statistical analysis. The data set of one group was used for the *development* and the data set of the other group for the *validation* of the prediction model. Statistical significance was accepted at the *p* < 0.05 level and a trend was identified at *p* < 0.10. IBM SPSS Statistics 25 and Microsoft Excel software were used for statistical analysis.

#### Prediction Model Development With Principal Component Analysis and Multiple Linear Regression

For the development of the prediction model, analyses were applied on the model *development* data set. Skin temperatures and skin heat fluxes measured at different body sites exhibited a linear relationship. Hence, the parameters could not be used directly to calculate a multiple linear regression model to predict rectal temperature, as this procedure requires uncorrelated parameters to avoid multicollinearity (Field, 2018). A principal component analysis (PCA) was therefore performed to reduce the number of 18 measured predictor parameters into a smaller set of uncorrelated components. Components with eigenvalues larger than Kaiser’s criterion of 1 were included. The resulting uncorrelated components were applied in a multiple linear regression analysis (forced entry method) to generate a first predictive equation for rectal temperature to address hypothesis 1 (*“Max-Input Model”*).

In order to improve practicality of the prediction model (hypothesis 3), a reduction of the prediction parameters was performed by removing one parameter of each pair of highly correlated parameters (*r* > 0.9). Thereby, the parameter showing the higher significance in a multiple regression analysis (stepwise method) was retained. This led to a set of uncorrelated parameters that were applied in a multiple linear regression analysis (forced entry method) and produced a first “reduced” predictive equation for rectal temperature. Subsequently, the number of parameters was further reduced in several steps by removing the least important parameters (=lowest standardized β value in regression analysis) one by one. This procedure was performed as long as the resulting prediction equations produced acceptable predictive values, i.e., standard error of the estimate (SEE) < 0.5°C (Gunga et al., 2009; Yokota et al., 2012; Niedermann et al., 2014b) and drop in *R^2^ adjusted* did not exceed 0.05 (*“Min-Input Model”*).

#### Prediction Model Validation

In order to validate the newly generated prediction equations, they were applied to the data sets of the participants that were put aside for *validation* purposes of the prediction model as stated above. The first prediction equation was used to address hypothesis 1, while the reduced prediction equations were used to address hypothesis 3. The same validation data set was also applied to the previously published prediction model from Niedermann et al. (2014b) to address hypothesis 2.

For the calculation of the component scores that are to be inserted in the prediction regression equation, the component score coefficients from PCA were used according to the following Equation 1 (*x*, measured value of parameter; $\bar{x}$, mean value of parameter; *SD*, standard deviation of parameter):

(1) $$component\;score=\sum coefficient_{parameter}\times \frac{x_{parameter}-{\bar{x}}_{parameter}}{SD_{parameter}}$$

To account for a potential systematic offset between the predicted and measured rectal temperature values, an average offset was calculated over all validation participants for the whole experimental duration and subtracted from the predicted value. These values can be added to the prediction equations when the models are applied elsewhere in data sets where no measure of rectal temperature is available. The validity of the prediction models was investigated by calculating the SEE and *R^2^ adjusted* from the measured and predicted rectal temperature to determine the quality of correspondence.

## Results

**FIGURE 4:**
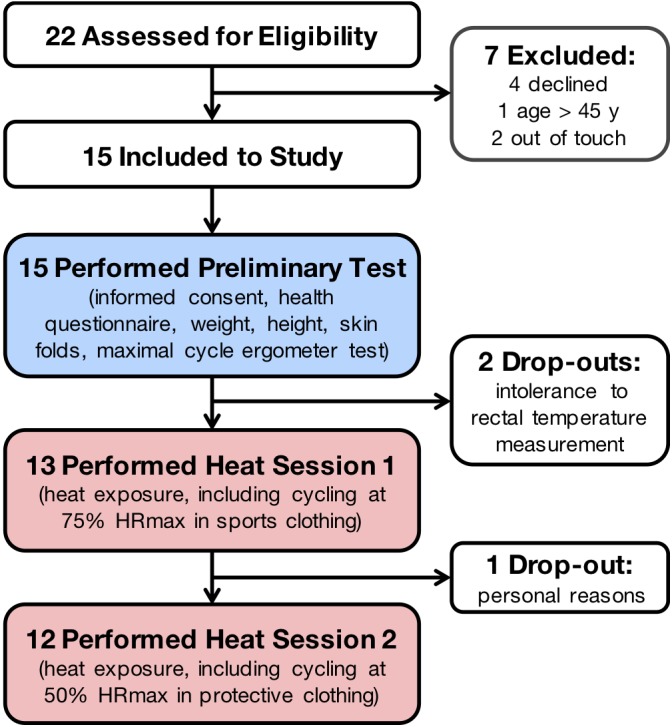
Flow diagram of the participants. HR max, maximal heart rate.

Twelve participants completed both heat sessions 1 and 2, while one participant completed only heat session 1 (Figure 4). Participants’ and experimental characteristics are shown in Table 1. After removal of sections with sensor malfunction, detachment, artifacts, and outliers, 10’048 time points (64% of the initially assessed 15’752 time points) with complete data for every parameter were retained for the development and validation procedure of the prediction model.

**Table 1:** Participants’ and experimental characteristics.

Variable	All participants	Prediction model development participants	Prediction model validation participants	Significance of difference between development and validation participants, *p*-value (two-tailed)
*N*	13	7	6	
Age, years	30. 9 (5.4)	32.6 (6.1)	28.9 (2.9)	0.227
Height, cm	179.2 (6.4)	180.6 (6.8)	177.5 (5.4)	0.424
Weight, kg	77.5 (6.1)	80.5 (6.4)	74.1 (3.0)	**0.058^t^**
BMI, kg/m^2^	24.1 (2.1)	24.6 (2.7)	23.4 (1.2)	0.411
Body fat, %	13.1 (4.3)	13.8 (4.0)	12.3 (4.6)	0.555
Sweat loss, l	0.9 (0.3)	0.9 (0.2)	1.0 (0.2)	0.231
**Cycling ergometer test:**				
Power max, W	273 (29)	267 (35)	280 (17)	0.452
HR max, beats/min	181 (9)	178 (9)	184 (8)	0.294
**Heat session 1 (75% HR max):**				
HR, beats/min	136 (7)	134 (8)	138 (6)	0.377
Power, W	155 (20)	168 (19)	118 (14)	**0.086^t^**
Duration, min	33.5 (8.8)	28.6 (9.0)	37.5 (5.0)	0.126
T rectal baseline, °C	37.1 (0.2)	37.0 (0.2)	37.0 (0.3)	0.810
T rectal max, °C	38.3 (0.2)	38.3 (0.3)	38.3 (0.1)	0.923
**Heat session 2 (50% HR max):**				
HR, beats/min	92 (3)	91 (2)	92 (4)	0.544
Power, W	52 (6)	53 (7)	50 (0)	0.363
Duration, min	43.7 (10.9)	39.4 (10.3)	49.6 (7.2)	**0.095^t^**
T rectal baseline, °C	36.9 (0.2)	36.9 (0.2)	37.0 (0.2)	0.336
T rectal max, °C	37.9 (0.3)	37. 9 (0.3)	37.9 (0.3)	0.906


*Data are numbers or means (± standard deviation in brackets). Bold values indicate trend or significance, **^*t*^***p* < 0.10 trend. BMI, body mass index; HR, heart rate.*

### Prediction Model Development With Principal Component Analysis and Multiple Linear Regression

#### “Max-Input Model”

Using the model *development* data set, PCA was conducted on the 18 non-invasive prediction parameters with orthogonal rotation (varimax). Sampling adequacy was verified with the Kaiser–Meyer–Olkin measure, KMO = 0.92, indicating “marvelous” according to Kaiser and Rice (1974), and all KMO values for individual items were > 0.77, which is well above the acceptable limit of 0.5 (Kaiser and Rice, 1974). Bartlett’s test of sphericity, χ^2^(153) = 179313.57, *p* < 0.001, indicated that correlations between items were sufficiently large for PCA. Two components had eigenvalues larger than Kaiser’s criterion of 1 and in combination explained 86.8% of the variance. The scree plot showed inflections that would justify retaining these two components. Table 2 shows the component loadings after rotation and component score coefficients. The items clustering on the same components indicate that component 1 represents skin temperature measures and component 2 skin heat flux measures, while heart rate contributed similarly to both components. Multiple linear regression analysis (forced entry method) was performed with the component score coefficients and produced a predictive equation for rectal temperature reported in Table 3.

**Table 2:** Rotated component loadings and component score coefficients for the “Max-Input Model”.

Parameter	Component 1	Component 2	Coefficient 1	Coefficient 2	Mean,$\bar{x}$
**Temperature non-insulated**					
T scapula	0.955		0.099	-0.061	35.69 (1.55)
T forearm	0.954		0.094	-0.047	34.86 (1.95)
T radial	0.921		0.115	-0.127	34.63 (2.11)
T thigh	0.917	0.309	0.073	0.016	34.87 (1.88)
T calf	0.914		0.075	0.010	33.91 (2.01)
T hand	0.914		0.096	-0.066	34.06 (2.53)
T arm	0.899		0.077	-0.001	35.21 (1.90)
T sternum	0.881	0.336	0.066	0.029	36.05 (1.41)
T rib	0.858	0.364	0.061	0.041	35.77 (1.37)
T ins rib	0.809	0.494	0.042	0.091	36.27 (1.40)
T forehead	0.803	-0.357	0.127	-0.206	35.10 (1.42)
**Temperature insulated**					
T ins radial artery	0.921		0.077	0.005	35.65 (1.96)
T ins scapula	0.901	0.379	0.064	0.042	36.52 (1.35)
T ins sternum	0.883	0.401	0.060	0.051	36.37 (1.32)
**Heart rate**					
HR	0.664	0.523	0.023	0.116	111.3 (34.1)
**Heat flux**					
HF scapula		0.905	-0.064	0.292	54.46 (70.88)
HF rib		0.856	-0.062	0.278	32.43 (42.65)
HF sternum		0.850	-0.088	0.299	18.03 (21.61)


*Only component loadings ≥ 0.3 are indicated. Mean values (±SD in brackets) are based on the model validation data. Units for mean values and SD are °C for T, beats/min for heart rate, and W/m^2^ for HF. HF, heat flux; HR, heart rate; ins, insulated; SD, standard deviation; T, temperature.*

**Table 3:** Regression equations for the two newly developed core body temperature prediction models.

Prediction model	Regression equation for prediction of rectal temperature
Max-Input Model	T = 0.2978 × factor score 1 + 0.2471 × factor score 2 + 37.2539
Min-Input Model	T = 0.0100 × Heart rate + 0.0837 × T ins scapula + 33.1735


*Ins, insulated; Max-Input Model, prediction model using all measured non-invasive parameters; Min-Input Model, prediction model using only the most relevant measured non-invasive parameters; T, temperature.*

#### “Min-Input Model”

A first reduction of the maximal number of 18 input parameters revealed seven uncorrelated parameters which were used to build a multiple linear regression equation based on the development data set. The seven included parameters comprised heart rate (standardized β = 0.527, *t* = 34.88, *p* < 0.001), insulated temperature at the scapula (standardized β = 0.246, *t* = 9.58, *p* < 0.001), non-insulated temperature at the radial artery (standardized β = -0.203, *t* = -11.76, *p* < 0.001), arm (standardized β = 0.201, *t* = 8.75, *p* < 0.001), and forehead (standardized β = 0.002, *t* = 0.13, *p* = 0.899), as well as heat flux at the sternum (standardized β = 0.025, *t* = 1.79, *p* = 0.074) and scapula (standardized β = 0.002, *t* = 0.111, *p* = 0.911). After stepwise explorative reduction of the number of parameters, the final regression equation that still produced acceptable predictive values, as defined in Section “Statistical Analysis”, included two parameters: heart rate (standardized β = 0.587, *t* = 40.54, *p* < 0.001) and insulated temperature at the scapula (standardized β = 0.232, *t* = 16.01, *p* < 0.001). The predictive regression equation from these two parameters is shown in Table 3. The prediction parameters used in the two newly developed prediction models (“Max-Input Model” and “Min-Input Model”) in comparison to the previously published model from Niedermann et al. (2014b) are listed in Table 4.

**Table 4:** Parameters used for the previously published and the two newly developed prediction models.

Parameter	Niedermann et al., 2014b	Max-Input Model	Min-Input Model
**Temperature non-insulated**			
T scapula		**X**	
T forearm	**X**	**X**	
T radial		**X**	
T thigh	**X**	**X**	
T calf		**X**	
T hand		**X**	
T arm	**X**	**X**	
T sternum		**X**	
T rib		**X**	
T ins rib		**X**	
T forehead		**X**	
**Temperature insulated**			
T ins radial artery		**X**	
T ins scapula		**X**	**X**
T ins sternum		**X**	
**Heart rate**			
HR	**X**	**X**	**X**
**Heat flux**			
HF scapula	**X**	**X**	
HF rib		**X**	
HF sternum	**X**	**X**	


*HF, heat flux; HR, heart rate; ins, insulated; Max-Input Model, prediction model using all measured non-invasive parameters; Min-Input Model, prediction model using only the most relevant measured non-invasive parameters; T, temperature.*

### Prediction Model Validation

**FIGURE 5:**
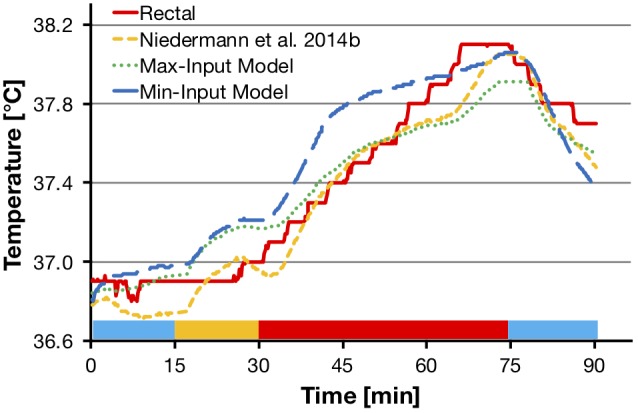
Comparisons of measured rectal temperature from heat session 1 (75% HR max cycling intensity, sports t-shirt and shorts) with the model from Niedermann et al. (2014b), the “Max-Input Model”, and the “Min-Input Model”, respectively. The graph shows a representative example from one participant. Colored bars at the bottom of the graph represent experimental phases as shown in Figure 2. HR max, maximal heart rate; Max-Input Model, prediction model using all measured non-invasive parameters; Min-Input Model, prediction model using only the most relevant measured non-invasive parameters; T, temperature.

**FIGURE 6:**
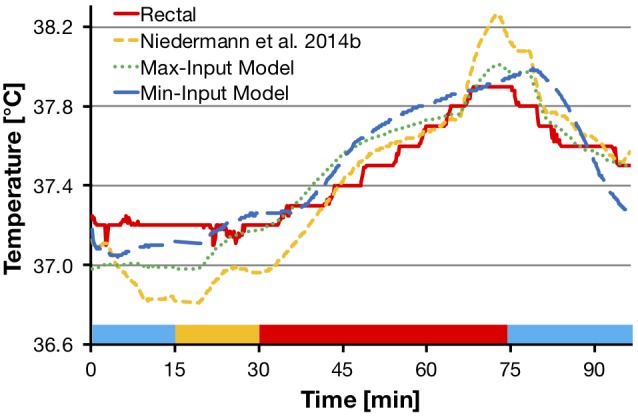
Comparisons of measured rectal temperature from heat session 2 (50% HR max cycling intensity, protective firefighter jacket and trousers) with the model from Niedermann et al. (2014b), the “Max-Input Model”, and the “Min-Input Model”, respectively. The graph shows representative example from the data of the same participant as in Figure 5. Colored bars at the bottom of the graph represent experimental phases as shown in Figure 2. HR max, maximal heart rate; Max-Input Model, prediction model using all measured non-invasive parameters; Min-Input Model, prediction model using only the most relevant measured non-invasive parameters; T, temperature.

*R^2^ adjusted* and SEE between the measured and predicted rectal temperature for the two newly developed prediction models were calculated based on the values of the *validation* data set and are reported in Table 5. A representative example of one participant’s measured and predicted rectal temperature during heat session 1 is depicted in Figure 5. Figure 6 illustrates these comparisons for heat session 2, also with example data from the same participant as in Figure 5. Average systematic offsets between the predicted and measured rectal temperature values of the validation participants were subtracted from the predicted values [offsets for the model from Niedermann et al. (2014b) = 0.24°C, “Max-Input Model” = -0.15°C, and “Min-Input Model” = -0.06°C, respectively].

**Table 5:** Validity of the previously published and the two newly developed prediction models.

Prediction model	*R^2^ adjusted*	SEE, °C
Niedermann et al., 2014b	0.708	0.276
Max-Input Model	0.703	0.278
Min-Input Model	0.677	0.290


*Acceptance criterion equals 0.5°C. Max-Input Model, prediction model using all measured non-invasive parameters; Min-Input Model, prediction model using only the most relevant measured non-invasive parameters; SEE, standard error of the estimate.*

## Discussion

This study *aimed* (1) to define the validity of a novel multi-parameter model to predict rectal temperature under two different exercise and clothing conditions in a hot and moderately humid environment, as well as at rest at normal room temperature; (2) to compare the novel prediction model with a previously published model (Niedermann et al., 2014b); and (3) to characterize the extent to which a stepwise reduction of the number of measured parameters, with the goal to improve practicality, affects the validity of the initial prediction model. The *main finding* was that we could generate a prediction model with only two input measurements (heart rate and insulated skin temperature at the scapula, “Min-Input Model”; SEE = 0.29°C, *R^2^* = 0.68) that had similar predictive power compared to two more comprehensive models. Of the two comprehensive models, the new model comprising all 18 input parameters (“Max-Input Model”; SEE = 0.28°C, *R^2^* = 0.70) did not outperform the predictive power of the previously developed model (Niedermann et al., 2014b) which included only six measures and no insulated skin temperatures (SEE = 0.28°C, *R^2^* = 0.71). Therefore, we conclude that the “Min-Input Model” provided comparable validity and superior practicality (only two measurement parameters) for estimating rectal temperature versus two other models requiring six or more input measures in young adult males.

The SEE of our two newly developed core body temperature prediction models (“Max-Input Model”, 0.28°C; “Min-Input Model”, 0.29°C) was substantially lower than the initially set acceptance criterion of < 0.5°C deviation from the measured rectal temperature. The models explained 70 and 68% of the variance in rectal temperature, respectively. These results confirm our *first hypothesis* that the proposed models deviated < 0.5°C from measured rectal temperature. Furthermore, the validity was also well in the range of previously reported prediction models. Richmond et al. (2015) presented a model which estimated rectal temperature with a SEE of 0.27°C and *adjusted R^2^* of 0.86, based on insulated skin temperature and microclimate temperature both measured at the lower neck, heart rate, and “work” (0 = rest, 1 = exercise). The model from Mazgaoker et al. (2017) applied a novel double sensor including heat flux measurements and performed within ±0.3°C of the rectal measurement. However, their prediction model consistently underestimated rectal temperature during exposure to exercise in the heat. A recent study developed an individualized mathematical prediction model using physical activity, heart rate, skin temperature at the chest, ambient temperature, and relative humidity as input measures and reported an average root mean squared error (RMSE) of 0.33°C compared to rectal temperature (Laxminarayan et al., 2018). Looney et al. (2018) presented a sigmoid prediction equation that relies exclusively on heart rate and predicted gastrointestinal temperature (collected by telemetric thermometer pill) with an RMSE of 0.26°C. Similarly, Buller et al. (2015) used heart rate as a single measure to estimate gastrointestinal temperature (RMSE = 0.22°C). Another recent publication combined skin temperature, heat flux, and heart rate to estimate gastrointestinal temperature using a Kalman filter (Welles et al., 2018). The authors found that measuring skin temperature and heat flux at the pectoralis, rib, or sternum resulted in similar prediction validity (RMSE = 0.18–0.20°C).

Our *second hypothesis*, that the additional inclusion of heat flux and insulated skin temperature measures improves prediction validity in comparison with our previous model (Niedermann et al., 2014b), has to be rejected as the new model did not perform better. A reason for this result might be found in the applied statistical analysis. For the development of both our previous model (Niedermann et al., 2014b) and the “Max-Input Model” a PCA was performed that reduced the large number of correlated parameters into a set of two uncorrelated components, representing either the skin temperature measures or the heat flux measures. Thereby, an additional benefit of the insulated skin temperatures for prediction validity, which is based on the assumption that this measure may closely represent core temperature (Richmond et al., 2015), could have been blunted. Nevertheless, the predictive potential of insulated skin temperature measure is supported by the outcome of the multiple regression analysis, which confirmed that insulated skin temperature at the scapula was the second most important predictor for rectal temperature. Similarly, a study by Richmond et al. (2015) found insulated skin temperature (at the lower part of the neck) to be the single most important physiological parameter in their prediction model.

The predictive validity of the “Min-Input Model”, which relies exclusively on the two most relevant predictors of the multiple linear regression analysis (heart rate and insulated skin temperature at the scapula), was only slightly lower than in the “Max-Input Model”. This finding confirms our *third hypothesis*, which stated that the validity of an adapted/reduced model still is in an acceptable range. This finding indicates the “Min-Input Model” as a good option in terms of practicality. Similar to our study, heart rate as a single measure or included into a predictive model was previously demonstrated to be a suitable predictor for core body temperature (Buller et al., 2015; Richmond et al., 2015; Laxminarayan et al., 2018; Looney et al., 2018; Welles et al., 2018). The predictive importance of heart rate is based on the relationship with metabolic activity (and consequently metabolic heat production) and heat transfer to the skin (via skin perfusion) (Buller et al., 2013; Niedermann et al., 2014b). During passive heat strain, an increase in skin blood flow contributes to the convective heat transfer to the extremities in order to increase surface area for dry and evaporative heat loss and results in a concomitant increase of heart rate. During exercise, however, heart rate does not only rise to promote cooling, but also to cover the additional oxygen needs. This makes the cardiovascular response to heat strain in combination with exercise complex to apportion (Richmond et al., 2015). Another important aspect for heart rate being a critical predictor of rectal temperature may be the direct effect of elevated temperature on the sinoatrial and atrioventricular cardiac nodal cells (Jose et al., 1970; Gorman and Proppe, 1984; Crandall and Wilson, 2015). Moreover, heart rate is increased in the heat due to altered autonomic nervous system activity, i.e., sympathetic activation and parasympathetic withdrawal (Gorman and Proppe, 1984; Crandall and Wilson, 2015). The inclusion of insulated skin temperature at the scapula in the “Min-Input Model” might be related to the proximal site of the temperature reading, which may better represent core body temperature in comparison to the more distal temperature measurement sites (e.g., calf, thigh, hand, and forearm). In addition, the placement of the insulated temperature sensor over a bony site (Richmond et al., 2013, 2015), i.e., over the inferior angle of the scapula in our study, may better reflect core body temperature compared to sites over muscle tissue (Brajkovic et al., 2006). Interestingly, heat flux data were not included in the new “Min-Input Model” contributing only 2.7% of explained variance if it were added to the reduced model, which is in contrast to our previous model (Niedermann et al., 2014b). The main issue in this study was the occurrence of a high number of measurement artifacts for the heat flux measurement data, thus reducing the predictive value of this parameter.

A methodological strength of this study was the integration of two different exercise and clothing conditions in a hot and moderately humid climate, as well as a resting and recovery condition at normal room temperature. This is promising in terms of developing prediction models that are applicable under different activity, clothing, and climatic scenarios. Notwithstanding, the following limitations should also be considered. The two new prediction models are valid for a population with similar characteristics as they were found in the participants of this study, e.g., fitness level, body composition, age, and sex. It has, however, been reported that gender differences in thermoregulation can be explained mainly through fitness level and body composition (Kaciuba-Uscilko and Grucza, 2001) and can be normalized by body weight and surface area (Gagnon and Kenny, 2012), and thus may play a limited role (Cheuvront, 2014; Cramer and Jay, 2014). The applicability of a general model for use on other cohorts requires further exploration. A further limitation might apply to the use of rectal temperature as the reference method representing core body temperature. Due to its thermal inertia and higher dependence on conductive heat transfer, rectal temperature responds more slowly in comparison to esophageal temperature (Taylor et al., 2014). This might explain why in the present study there are larger separations between actual rectal temperature and predicted values in transient conditions (i.e., at the beginning of the heat exposure and at the end of the final rest period) as can be seen in Figures 5, 6. It appears that toward the end of exercise, the difference between predicted and actual values becomes smaller. Therefore, one might expect the prediction to be more accurate during stable conditions where heat balance is achieved. Despite the common use of rectal and gastrointestinal temperatures in laboratory experiments and model development, the use of more responsive methods, like esophageal temperature, is warranted for investigation in future models.

## Conclusion

The present study provides two novel prediction models for core body temperature that were validated in a hot and moderately humid environment under different exercise and clothing conditions, as well as at normal room temperature at rest. Thereby, one of the two new prediction models is based exclusively on the two measurement parameters heart rate and insulated skin temperature at the scapula (“Min-Input Model”; SEE = 0.29, *R^2^* = 0.68). These parameters were found to be the two most relevant parameters for the prediction of rectal temperature, among 18 assessed non-invasive parameters. The other, more complex model developed in this study, included the maximal input of all 18 non-invasive parameters (“Max-Input Model”; SEE = 0.28°C, *R^2^* = 0.70), but only performed marginally better than the “Min-Input Model” and similar to a previously developed model from Niedermann et al. (2014b) (SEE = 0.28°C, *R^2^* = 0.71). Therefore, we conclude that the “Min-Input Model” provided comparable validity and superior practicality (only two measurement parameters) for estimating rectal temperature in young adult males, versus two other models requiring six or more input measures. As a subsequent step, the latter model should be investigated in more diverse populations (e.g., females, older adults, patients), as well as in other exercise modalities, clothing, and climatic conditions, in order to verify its general validity.

## Data Availability Statement

The raw data supporting the conclusions of this manuscript will be made available by the authors, without undue reservation, to any qualified researcher.

## Author Contributions

BM, RR, and SA contributed conception and design of the study. PE recruited the participants. PE and SK conducted the experimental sessions. MB, PE, and SK executed data processing. PE and MB performed the statistical analysis. MB, PE, RR, and SA contributed to data interpretation. PE wrote the first draft of the manuscript. All authors contributed to manuscript revision, read and approved the submitted version.

## Conflict of Interest Statement

The authors declare that the research was conducted in the absence of any commercial or financial relationships that could be construed as a potential conflict of interest.
